# Effects of glial cell line-derived neurotrophic factor on cultured murine retinal progenitor cells

**Published:** 2010-12-31

**Authors:** Jinmei Wang, Jing Yang, Ping Gu, Henry Klassen

**Affiliations:** 1 Gavin Herbert Eye Institute and Stem Cell Research Center, University of California, Irvine, CA; 2 Department of Ophthalmology, Shanghai Ninth People's Hospital, School of Medicine, Shanghai Jiaotong University, Shanghai, China

## Abstract

**Purpose:**

Glial cell line-derived neurotrophic factor (GDNF) is neuroprotective of retinal neurons, and transduced retinal progenitor cells (RPCs) can deliver this cytokine for the treatment of retinal diseases, yet the potential effects of GDNF on RPCs have received little attention.

**Methods:**

Murine RPCs were assessed under multiple conditions in the presence or absence of epidermal growth factor (EGF, 20 ng/ml) and/or GDNF (10 ng/ml) using a variety of techniques, including live-cell imaging, caspase-3 activity assay, whole genome microarray, quantitative polymerase chain reaction (qPCR), and western blotting.

**Results:**

Live monitoring revealed that formation of initial aggregates resulted largely from the collision and adherence of dissociated RPCs, as opposed to clonal proliferation. Spheres enlarged in size and number, with more reaching the threshold criteria for cross-sectional areas in the EGF+GDNF condition. Proliferation was measurably augmented in association with EGF+GDNF, and *K_i_-67* expression was modestly increased (1.07 fold), as were hairy and enhancer of split 5 (*Hes5*), mammalian achaete-scute homolog 1 (*Mash1*), and *Vimentin*. However, global gene expression did not reveal a notable treatment-related response, and the expression of the majority of progenitor and lineage markers examined remained stable. GDNF reduced RPC apoptosis, compared to complete growth-factor withdrawal, although it could not by itself sustain mitotic activity.

**Conclusions:**

These data support the feasibility of developing GDNF-transduced RPCs as potential therapeutic agents for use in retinal diseases.

## Introduction

The neural retina is subject to a range of degenerative conditions, including those like retinitis pigmentosa and age-related macular degeneration that involve photoreceptor loss. Similar to other compartments of the central nervous system, the mammalian retina lacks the capacity to regenerate following injury, and there are at present no restorative therapies available for diseases involving photoreceptor loss. Retinal progenitor cells (RPCs) actively generate the mature neurons and Müller glia of the neural retina during eye development. A large body of evidence has now shown that RPCs can be isolated, expanded [1-4], and transplanted in various animal models [1,5-9]. The transplantation of RPCs to the abnormal retina has become an important strategy in retinal regeneration research.

Studies on avian and mammalian embryos have identified factors that influence the behavior of RPCs during normal retinal development and in response to injury. Particular interest has been directed toward the subset of factors with demonstrated neuroprotective efficacy because of their potential clinical relevance. One important effective molecule known to rescue retinal neurons, including photoreceptors, in multiple animal models is the glial cell line-derived neurotrophic factor (GDNF) [10-12]. GDNF is a distant member of the transforming growth factor-β family of growth factors and a member of the GDNF family, which also includes neurturin, persephin, and artemin [13]. GDNF was originally purified and characterized in 1993 and found to be a survival factor for embryonic dopaminergic midbrain neurons in culture [14]. It later became clear that GDNF also acts as a potent neurotrophic factor in a variety of other contexts. GDNF is widely distributed in the developing central nervous system and has been confirmed to subserve pivotal roles in many additional tissues, including the peripheral nervous system, inner ear, embryonic kidney, gastrointestinal tract, skeletal muscle [15-17] and spermatogonial stem cells [18].

In the past few years, the potential of GDNF as a therapeutic agent for the treatment of neurodegenerative conditions such as Parkinson disease, which is characterized by dopaminergic cell loss, has been actively explored [19,20]. In the eye, it has been shown that both GDNF and its receptors are synthesized in the retina [15,21], thereby suggesting that this factor has an innate neurotrophic role in this tissue. Several lines of work, using recombinant protein, knockout, or overexpression methods, have implicated GDNF in the regulation of cell-cycle progression, specifically in terms of promoting neuroblast proliferation at early stages of development, as well as promoting the survival and differentiation of retinal photoreceptors [22,23]. In addition to these roles, GDNF may also delay the onset of apoptosis and participate in the regulation of cellular migration, although in this regard the literature is more variable. For instance, Clarkson and colleagues [24] showed that GDNF had no discernable effect on apoptosis in astrocytes derived from the embryonic mesencephalon or from the neonatal cortex. Iwashita and colleagues [25] also showed that GDNF did not affect the survival or proliferation of neural crest stem cells. Together, these results indicate that although the neurotrophic role is well established, the effects of GDNF do not entirely generalize across cell types or developmental stages.

GDNF has shown considerable potential as a novel therapeutic agent for the treatment of neurodegenerative diseases, including those of the retina; however, a significant challenge to implementation has been the need for sustained local delivery over lengthy time periods. An attractive strategy for neutrophic factor delivery that has been considered is the transplantation of stem or progenitor cells that have been genetically modified to overexpress the particular gene of interest [26,27]. When contemplating such a strategy, it is important to consider the impact of the delivered factor on the behavior of the cell used for delivery. In addition, it is of interest to know how the factor might influence tissue-specific stem cells residing within the recipient host tissue. At present, little is known regarding the influence of GDNF on cultured RPCs. Here we investigate the influence of GDNF on a previously characterized and transplantable population of mitotically active progenitor cells derived from the immature murine retina.

## Methods

### Isolation and culture of late retinal progenitor cells

RPCs were previously isolated from the neural retina of postnatal day 1GFP transgenic mice [28]. Briefly, retinas were harvested from newborn GFP transgenic C57BL/6 mice (gift from Masaru Okabe, University of Osaka, Osaka, Japan) [29] and subjected to several cycles of collagenase digestion to dissociate the tissue. Cells were then forced through a nylon mesh of 100 µm pore size, centrifuged, and resuspended in standard culture medium, containing Dulbecco’s modified Eagle’s medium/Ham’s F12 1:1 (Omega Scientific, Tarzana, CA) supplemented containing 1% (by vol.) N2 neural supplement (Gibco, Invitrogen, Carlsbad, CA), 2 mM L-glutamine (Sigma Aldrich, St. Louis, MO), 2,000 U nystatin (Gibco), 50 mg/ml penicillin-streptomycin (Sigma), and 20 ng/ml recombinant human epidermal growth factor (EGF; Invitrogen, Carlsbad, CA). GFP+ neurospheres appeared within the first 2 to 3 days. Culture media were changed every 2 days, and proliferating cells were passaged at regular intervals of 4–5 days. These cells were immunoreactive for nestin (a marker for neural progenitor cells) and *K_i_-67* (a marker for cell proliferation).

### Treatment conditions

**Figure 1:**
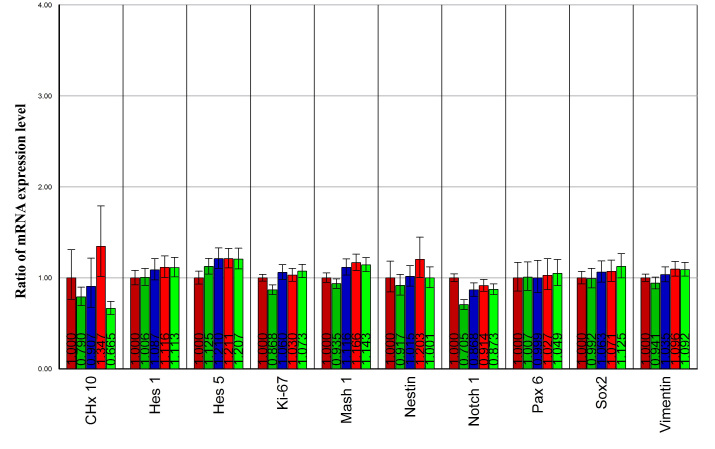
Gene expression profile of retinal progenitor cell progenitor markers under different concentrations of glial cell line-derived neurotrophic factor treatment. Cultured genetically modified (GFP+) RPCs were treated with 2.5, 5, 10, and 20 ng/ml of glial cell line-derived neurotrophic factor (GDNF) for 5 days, then RNA was isolated, and quantitative polymerase chain reaction (qPCR) assay was performed. The progenitor markers in those samples remained at similar levels under different concentrations of GDNF. In each gene column, the five bars from left to right represent cells in epidermal growth factor (EGF) alone (dark red), in EGF+2.5 ng/ml GDNF (dark green), in EGF+5 ng/ml GDNF (blue), in EGF+10 ng/ml GDNF (red), and in EGF+20 ng/ml GDNF (green).

To first determine the optimal concentration of GDNF for use in the present study, we tested the effects of four different concentrations of GDNF (2.5, 5, 10, and 20 ng/ml) on the gene expression profile of genetically modified RPCs. Cells were treated with GDNF for 5 days, RNA was extracted, and a quantitative PCR (qPCR) assay was performed. The progenitor markers remained at similar levels to those of untreated controls under the different concentrations of GDNF examined (Figure 1). Given this general equivalence, combined with our interest in looking for a possible physiologic (as opposed to toxic) influence, we chose 10 ng/ml for use in our system.

RPCs were cultured in standard base media with the only difference being the presence or absence of EGF (20 ng/ml) and/or 10 ng/ml recombinant human GDNF (PHC7045; Biosource, Invitrogen) as follows:

1) No recombinant factors (nonmitogenic conditions, negative control),

2) EGF alone (proliferation conditions, positive control, i.e., identical to “standard medium”),

3) GDNF alone, or

4) EGF+GDNF together.

### Morphometry

Cellular morphology was recorded on treatment day 1, 3, and 5 using a microscope-mounted camera and imaging system. In addition, cellular proliferation and sphere formation were also recorded and quantified every 45 min using an IncuCyte live-cell imaging system (Essen Instruments, Ann Arbor, MI) located within the incubator.

### Cell viability

RPCs were cultured under four different conditions consisting of the base medium and either no added growth factors, EGF alone, GDNF alone, or EGF+GDNF, for 5 days. Cell viability was measured using a Cell Counting Kit-8 assay (Dojindo, Kumamoto, Japan). In brief, cells were suspended at a final concentration of 1×10^4^ cells/well and cultured in 96-well, flat-bottomed microplates. At the end of each treatment day, 10 μl WST-8 [2-(2-methoxy-4-nitrophenyl)-3-(4-nitrophenyl)-5-(2,4-disulfophenyl)-2H-tetrazolium, monosodium salt] was added to each well. The plates were incubated for an additional 4 h at 37 °C to convert WST-8 into formazan. The absorbance was then measured at 450 nm with a spectrophotometer. Cell viability is in direct proportion to the absorbance at 450 nm; therefore, viability was expressed as the A_450_ value. All experiments were performed in 96-well plates (ten wells/condition).

In addition, growth of RPCs in medium with EGF alone, or with EGF and GDNF, was assessed over a one-month time course. Cells were passaged before reaching confluence. At each passage, the cell number was counted using a hemocytometer, and a population doubling level (PDL) was determined as current PDL=Log(final harvest cell number/initial seeded cell number)×3.33.

### Caspase-3 activity assay

After 24 h culture, RPCs exposed to the four different conditions mentioned above were collected and washed in cold phosphate buffered saline (PBS; 2.68 mM KCI, 1.47 mM KH_2_PO_4_, 135.60 mM NaCl, 8.10 mM Na_2_HPO_4_). Cell pellets were resuspended in 100 μl lysis buffer. After 20 min incubation on ice, each cell lysate was spun at 18,000× g at 4 °C for 15 min. The supernatant was collected and 5 μl of cell lysate from each condition was diluted to 100 μl in the assay buffer consisting of 20 mM Hepes at pH 7.4, 0.1% CHAPS, 5 mM dithiothreitol, and 2 mM EDTA, supplemented with 2 mM of the caspase-3 substrate Ac-DEVD-pNA. The enzymatic activity was determined spectrophotometrically at 405 nm (molar absorptivity of p-nitroaniline, ε^mM^=10.5 at 405 nm). Caspase-3 activity was determined as µmol pNA released per minute per milligram of protein.

### RNA isolation

RPCs were grown in culture media that contained EGF or EGF+GDNF for 5 days. Total RNA was extracted from each sample using the RNeasy Mini Kit (Cat. No. 74104; Qiagen, Valencia, CA). After RNA isolation, samples were treated by DNaseI (Macherey-Nagel, Düren, Germany) to digest and eliminate any contaminating genomic DNA. RNA concentration was measured for each sample at a wavelength of 260 nm (A_260_), and the purity of extracted total RNA was determined by the A_260_/A_280_ ratio. Quantitative reverse-transcriptase PCR analyses and microarray analysis were only performed on samples with A_260_/A_280_ ratios between 1.9 and 2.1.

### Microarray processing and analysis

After RNA isolation (two treatments, with three samples per treatment), 1 μg total RNA from each sample was used to prepare material for hybridization with each of the six Affymetrix mouse gene 1.0 ST arrays at the UCI Genomics High-Throughput Facility at the University of California, Irvine, as recommended by the manufacturer (Affymetrix Genechip Whole Transcript Sense Target Labeling Assay Manual; Affymetrix, Inc., Santa Clara, CA). The integrity and concentration of total RNA were measured, followed by hybridization, scanning, and generation of raw expression data, which were subsequently normalized using a standard technique by the same facility, as follows.

All starting total RNA samples were quality assessed before beginning the target preparation/processing steps by running out a small amount of each sample (typically 25–250 ng/well) onto a RNA 6000 Nano LabChip that was evaluated on an Agilent Bioanalyzer 2100 (Agilent Technologies, Palo Alto, CA). Single-stranded, then double-stranded, cDNA was synthesized from the poly(A)^+^mRNA present in the isolated total RNA (typically 100 ng total RNA starting material for each sample reaction) using the GeneChip WT cDNA Synthesis Kit (Affymetrix, Inc., Santa Clara, CA) and random hexamers tagged with a T7 promoter sequence. The double-stranded cDNA was then used as a template to generate many copies of antisense cRNA from an in vitro transcription reaction for 16 h in the presence of T7 RNA polymerase using the Affymetrix Genechip WT cDNA Amplification Kit. Ten micrograms of cRNA were input into the second-cycle cDNA reaction with random hexamers that were used to reverse-transcribe the cRNA from the first cycle to produce single-stranded DNA in the sense orientation.

The single-stranded DNA sample was fragmented (WT Terminal Labeling Kit, Affymetrix) to an average strand length of 60 bases (range 40–70 bp) following prescribed protocols (Affymetrix GeneChip WT Sense Target Labeling Assay Manual). The fragmented single-stranded DNA was subsequently labeled with recombinant terminal deoxynucleotidyl transferase and the Affymetrix proprietary DNA Labeling Reagent, which is covalently linked to biotin. Following the recommended procedure, 0.54 μg of this fragmented single-stranded target cDNA was hybridized at 45 °C with rotation for 17 h (Affymetrix GeneChip Hybridization Oven 640) to probe sets present on an Affymetrix mouse-gene 1.0 ST array. The GeneChip arrays were washed and then stained (streptavidin-phycoerythrin) on an Affymetrix Fluidics Station 450 (Fluidics protocol FS450_007). Arrays were scanned using the GeneChip Scanner 3000 7G and GeneChip Operating Software v1.4 to produce CEL intensity files.

Normalization was performed using the probe logarithmic intensity error (PLIER) estimation method, which includes a quantile normalization protocol within the associated software algorithm. Briefly, the probe cell intensity files (*.CEL) generated above were analyzed using Affymetrix Expression Console software v1.1 using the PLIER algorithm to generate probe-level summarization files (*.CHP). The algorithm used was from PLIER v2.0 (quantification scale: linear; quantification type: signal and detection p value; background: PM-GCBG; normalization method: sketch-quantile).

Statistical analysis was subsequently conducted using JMP Genomic 3 software (SAS Institute Inc.) The raw data were log-2 transformed and imported into the software for analysis. The strategy used was to compare the gene expression profile of the control group (EGF alone) with the experimental group (EGF+GDNF). Data from the arrays were analyzed by the clustering of differences between treatments and identification of significant changes. Changes were considered statistically significant if the difference in expression between the groups had a p value less than 0.05.

### Reverse transcription and quantitative PCR

To further assess expression changes, transcription levels of selected genes were examined by performing semiquantitative real-time PCR using an Applied Biosystems 7500 Fast Real-Time PCR Detection System (Applied Biosystems, Foster, CA). Two micrograms of total RNA in a 20 μl reaction volume were reverse-transcribed using an Omniscript cDNA Synthesis Kit (Qiagen). Oligonucleotide primer sequences (Table 1) were designed using Primer 3 software. The primers were synthesized commercially (Invitrogen) and qPCR was performed in 20 μl total volume containing 10 μl of 2× Power SYBR Green PCR Master Mix (Applied Biosystems), 10 μl of cDNA, and 300 nM of gene-specific primers. Cycling parameters for qPCR were as follows: initial denaturation at 95 °C for 10 min, followed by 40 cycles of 15 s at 95 °C and of 1 min at 60 °C. To normalize template input, the β-actin transcript level was measured for each sample (endogenous control). The efficiency of the PCR reaction was measured with primers using serial dilution of cDNA (1:1, 1:5, 1:25, 1:125, 1:625, and 1:3,125). The relative expression of the gene of interest, (Etarget) ΔCt target (control-treated)/(Eref)ΔCt ref (control-treated) [30], was then evaluated by the Pfaffl method [30]. The value obtained for crossing threshold (Ct) represents the number of PCR cycles at which an increase in fluorescence signal (and therefore cDNA) can be detected above the background. The increase is exponential for the particular gene. Data are expressed as fold-change relative to untreated controls, after normalizing to β-actin (our choice from among a set of “house-keeping” genes).

**Table 1:** Primers used for quantitative RT–PCR.

**Genes**	**Accession number**	**Forward (5′-3′)**	**Reverse (5′-3′)**	**Annealing temperature (°C)**	**Product size (base pairs)**
*Nestin*	NM_016701	aactggcacctcaagatgt	tcaagggtattaggcaagggg	60	235
*Vimentin*	NM_011701	tggttgacacccactcaaaa	gcttttggggtgtcagttgt	60	269
*Sox2*	NM_011443	cacaactcggagatcagcaa	ctccgggaagcgtgtactta	60	190
*Hes1*	NM_008235	cccacctctctcttctgacg	aggcgcaatccaatatgaac	60	185
*Hes5*	NM_010419	caccgggggttctatgatatt	caggctgagtgctttcctatg	60	180
*Pax6*	NM_013627	agtgaatgggcggagttatg	acttggacgggaactgacac	60	132
*Chx10*	NM_007701	caatgctgtggcttgcttta	cttgagagccactgggctac	60	157
*Notch1*	NM_008714	acccactctgtctcccacac	gcttccttgctaccacaagc	60	123
*Mash1*	NM_008553	tctcctgggaatggactttg	ggttggctgtctggtttgtt	60	142
*K_i_-67*	X82786	cagtactcggaatgcagcaa	cagtcttcaggggctctgtc	60	170
*β3-tubulin*	NM_023279	cgagacctactgcatcgaca	cattgagctgaccagggaat	60	152
*DCX*	NM_010025	tgtaaactaaaacaaagacccgaag	aagtacctcacaagtcaaagaatgg	60	187
*Map2*	NM_001039934	agaaaatggaagaaggaatgactg	acatggatcatctggtaccttttt	60	112
*Recoverin*	NM_009038	atggggaatagcaagagcgg	gagtccgggaaaaacttggaata	60	179
*Rhodopsin*	NM_145383	tcaccaccaccctctacaca	tgatccaggtgaagaccaca	60	216
*PKC-α*	NM_011101	cccattccagaaggagatga	ttcctgtcagcaagcatcac	60	212
*CRALBP*	NM_020599	agggtctttgttcacggagat	tgccactagagcgttcctaaa	60	297
*GFAP*	NM_010277	agaaaaccgcatcaccattc	tcacatcaccacgtccttgt	60	184
*β-actin*	NM_007393	agccatgtacgtagccatcc	ctctcagctgtggtggtgaa	60	152
*c-myc*	NM_010849	gctgtagtaattccagcgagaga	aagttccagtgagaagtgtctgc	60	239
*Nanog*	NM_028016	ttggttggtgtcttgctcttt	caggaagacccacactcatgt	60	196
*SDF1*	NM_021704	gtattgtagctttccggtgtcag	aggaggtttacagcatgaaacaa	60	120
*CXCR4*	NM_009911	ctgtgtgatggtttgtttggtt	ttctaccaccatttcaggcttt	60	101
*Annexin V*	NM_009673	tgctcaggagtttaagactctgttt	taatctcggtcaatactttctcgtc	60	182
*Caspase 1*	NM_009807	acacgtcttgccctcattatct	gcagcaaattctttcacctctt	60	176
*Caspase 3*	NM_009810	aaggagcagctttgtgtgtgt	cgcctctgaagaagctagtca	60	107
*P4HB*	NM_011032	gcagcagaggctattgatgac	atcttcggagctgtctgttca	60	221
*FSP1*	NM_011311	acttccaggagtactgtgtcttcc	aaactacaccccaacacttcatct	60	128
*KLF4*	NM_010637	ctgaacagcagggactgtca	gtgtgggtggctgttctttt	60	218

### Western blotting

Samples for protein analyses were homogenized in CelLytic M (C2978; Sigma), and proteins were measured using the Bio-Rad detergent-compatible protein assay (500–0006; Bio-Rad). Proteins (50 µg) were electrophorised using a 3%–8% Tris-acetate gel (EA0375BOX; Invitrogen) for 70 min at 150 V and then transferred from the gel to a polyvinyldifluoride membrane using an iBot Gel Transfer Device (IB1001; Invitrogen). Western blotting was then performed using the WesternBreeze Chromogenic Western Blot Immunodetection Kit (WB7103, Invitrogen) according to the manufacturer’s protocol. After blockade of nonspecific binding sites using Blocker/Diluent solutions provided, the polyvinylfluoride membranes were incubated for 2 h at room temperature with mouse primary antibodies directed against either *K_i_-67* (556003, 1:200; BD), nestin (611658, 1:200; BD), or β-actin (A5441, 1:200; Sigma). This was followed by washing and 1 h incubation with an alkaline phosphatase-conjugated antimouse IgG secondary antibody provided in the kit. Immunoblots were developed using chromogenic substrate for 10 min. The reactive bands were scanned and quantified by densitometry using UN-SCAN-IT Gel 6.1 (Silk Scientific, Inc. Orem, UT) The density of product from the EGF treatment group was defined as 1, and the data from the EGF+GDNF condition was expressed as the fold-change of this value.

### Statistical analysis

The results represent the average of three experiments (±SE). Except where specifically indicated, each experiment was performed in triplicate. For cell viability and caspase-3 activity studies, ten fields per sample were analyzed for each condition. Statistical significance was determined by Student’s two-tailed *t*-test.

## Results

### RPC morphology and sphere formation

**Figure 2:**
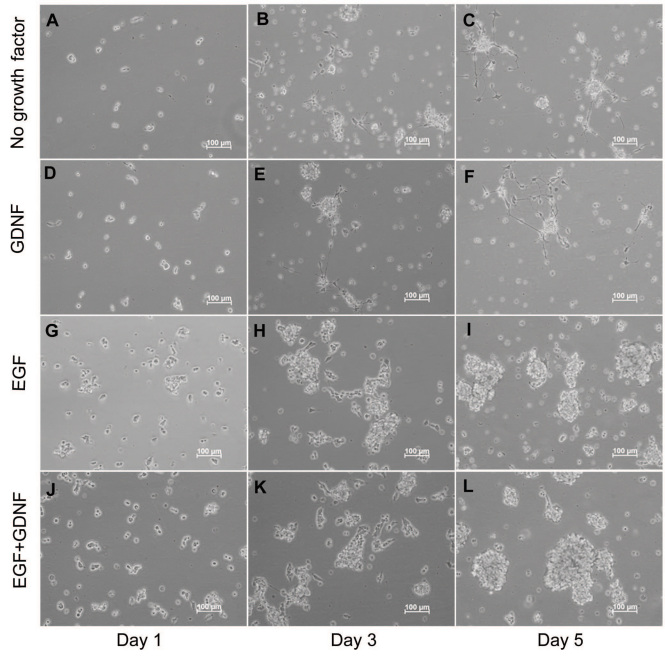
Changes in retinal progenitor cell morphology under different culture conditions. Retinal progenitor cell s were cultured in the same serum-free base media, but under four different treatment conditions defined by the presence or absence of added growth factors, as follows: 1) no growth factor (**A**-**C**), 2) glial cell line-derived neurotrophic factor (GDNF) alone (**D**-**F**), 3) epidermal growth factor (EGF) alone (**G**-**I**), and 4) EGF+GDNF (**J**-**L**). In each case, EGF was used at a final concentration of 20 ng/ml and GDNF at 10 ng/ml. The morphology of cells in each condition was assessed on day 1, 3, and 5. Increased extension of processes appeared in the “no growth factor” group (**A**-**C**; **D**-**F**), with similar changes observed in the “GDNF alone” group (**A**-**C**; **D**-**F**). Cells grown in EGF+GDNF appeared to form more and larger spherical cellular aggregates (spheres) over the course of 5 days. Magnification was ×100. Scale bars: 100 μm.

RPCs were subjected to different treatment conditions for up to 5 days. As a result, the morphology of RPCs clearly differed in response to the presence or absence of EGF (Figure 2). There was increased extension of processes in response to the absence of EGF (Figure 2A-F), whereas the appearance of undifferentiated RPCs cultured in either EGF alone or EGF+GDNF remained stable throughout the treatment period. Undifferentiated RPCs either adhered to the surface of the uncoated flasks or were observed floating in the culture medium, either as single cells or spheres of different sizes. From day 3, short processes with few, if any, branches extended from some of the adherent cells (Figure 2H,K). No obvious morphological differences were observed in response to the presence of 10 ng/ml GDNF (GDNF alone) when compared to culture conditions without EGF (no recombinant factors; Figure 2D-F). However, in the presence of GDNF (EGF+GDNF), more and larger spheres were observed, as compared to EGF alone (Figure 2I,L).

**Figure 3:**
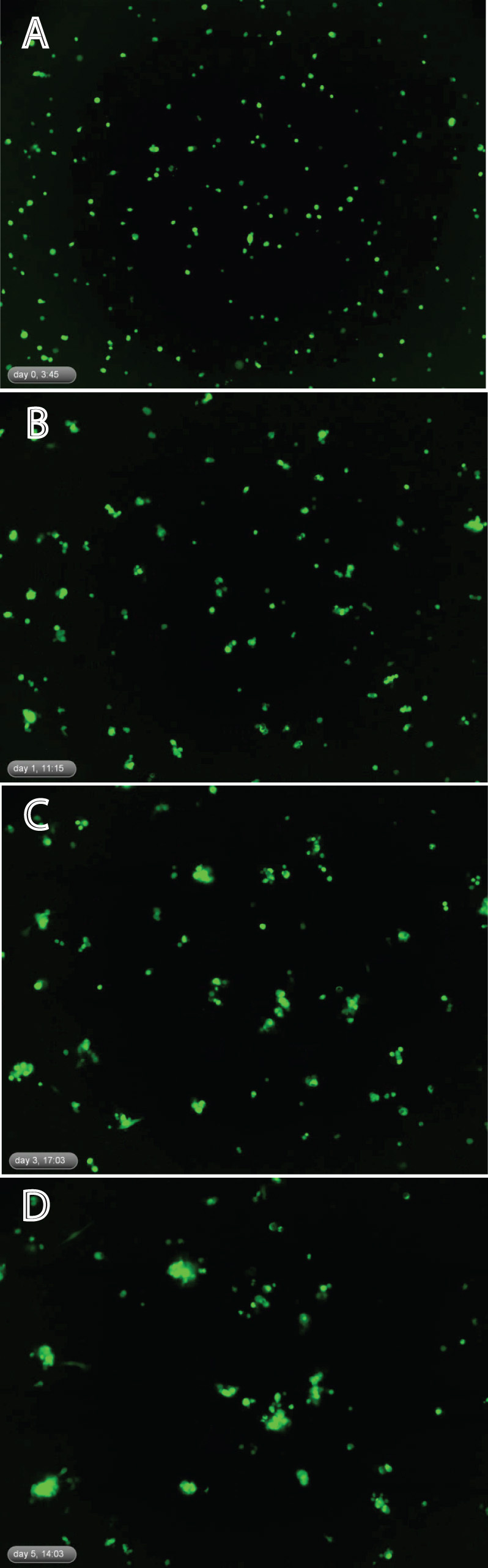
Still frames taken at the following time points in culture: **A**. Day 0, 3:45 h; **B**. Day 1, 11:15 h; **C**. Day 3, 17:03 h; **D**. Day 5, 14:03 h. These images highlight the basic findings of the time-lapse experiment, namely, the simultaneous enlargement of focal aggregates (proto-spheres) together with the progressive decrease in the overall number of aggregation centers. In the video this can be seen to largely correspond to the adherence of cells and cellular clusters over time.

**Figure 4:**
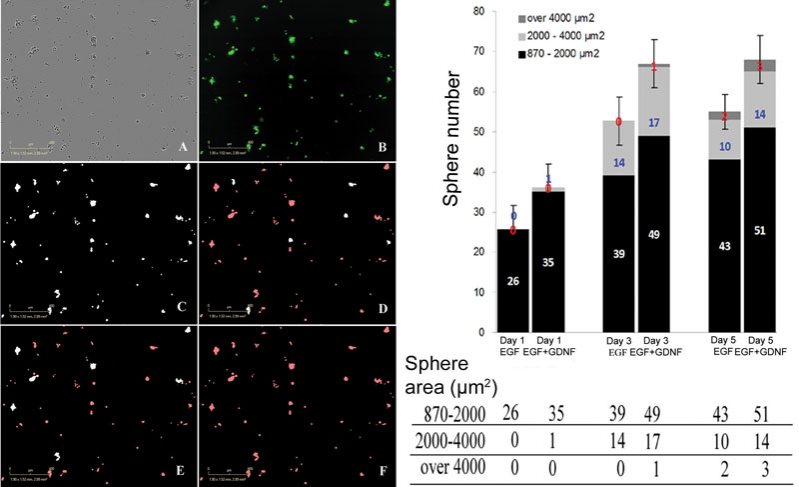
Quantitative analysis of sphere formation under different culture conditions. The left panel shows phase (**A**) and fluorescent (**B**) photomicrographs of murine retinal progenitor cells (RPCs). Examples of object thresholding and quantification using image analysis software are shown (**C**-**F**), with white indicating selected objects and red indicating rejected objects. Specifically, a given microscopic field is thresholded to select all spheres (**C**), small spheres (**D**), middle-sized spheres (**E**), and large spheres (**F**; none present in this image). The scale bar is 400 µm. Right panel: RPCs were cultured in medium containing epidermal growth factor (EGF) or EGF + glial cell line-derived neurotrophic factor (GDNF) for 5 days. Number and cross-sectional area of spherical cellular aggregates (spheres) larger than 870 µm^2^ are shown for each time point. Spheres meeting threshold criteria increased in number along the time course in both conditions. Significantly greater numbers and larger area of the spheres were found in the EGF+GDNF condition, compared to EGF at day 5, with the earlier trend seen at day 1 and day 3 not reaching statistical significance by the criterion used. Data represent the mean of six samples from same plating (*p<0.05). x-axis shows different medium conditions at day 1, 3, and 5; y-axis shows the number of spheres. Different shading within the histogram shows sphere area (µm^2^). Black is for small spheres, light gray for middle-sized, and dark gray for large. Standard deviation was used to generate the error bars, which reflect the sphere numbers.

The proliferation of RPCs and formation of spherical cellular aggregates (spheres) were also monitored via the IncuCyte system over the five-day time course, allowing comparison of EGF and EGF+GDNF conditions. By way of the acquired video, dissociated RPCs were seen to exhibit a strong propensity to aggregate in culture, with the formation of spheres being evident as early as 5.5 h after seeding. Aggregates started with the movement of suspended single RPCs within the culture medium and adherence to other cells, as opposed to cell division and clonal expansion (see Appendix 1, Figure 3). Over time, the spheres enlarged and their movement lessened, consistent with an increased contribution of cell proliferation to sphere formation. The number of spheres with a cross-sectional area of >870 µm^2^ increased in both treatment conditions, beginning on day 0 and continuing to day 5, at which time greater numbers of spheres had reached threshold criteria in the GDNF-supplemented medium (p<0.05). In addition, more spheres had attained higher categories of cross-sectional area in this treatment condition as well, consistent with increased cellular proliferation (Figure 4).

### Cell viability and proliferation

**Figure 5:**
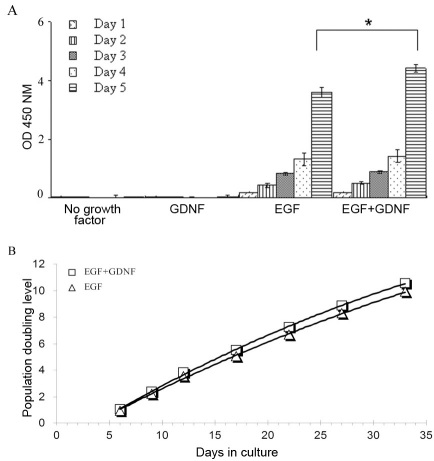
Viability of murine retinal progenitor cells (RPCs) in different culture media. **A**: Murine RPCs exhibited exponential growth in the presence of epidermal growth factor (EGF) alone (20 ng/ml), as well as with EGF+Glial cell line-derived neurotrophic factor (GDNF; 20 and 10 ng/ml, respectively), but diminished from day 1 in the absence of both factors, or with GDNF alone. A difference in the surviving cell number between the EGF-containing groups was observed, with the addition of GDNF to the medium conferring a statistically significant advantage over EGF alone at day 5. This effect was not evident at earlier time points. The y-axis shows absorbance at 450 nm. All experiments were performed in 96-well plates (ten wells/condition). Error bars show standard deviation (SD), * indicates p<0.03. **B**: Growth of RPCs over a one-month period in EGF or EGF+GDNF was monitored by manual cell counts (with a hemocytometer). The growth curves thus obtained confirmed that the addition of GDNF (10 ng/ml) to standard EGF-containing medium does not impair the proliferation of murine RPCs and in fact provides a detectible advantage.

Murine RPCs exhibited exponential growth in the presence of EGF alone, as well as with EGF+GDNF, but diminished asymptotically in the absence of both factors, or with GDNF alone. Cell viability was compared for the EGF alone versus the EGF+GDNF conditions using the cell-counting kit. By this method, the number of RPCs increased from day 1 to 4, although there was no difference between conditions. By day 5, however, there were significantly more viable cells in the EGF+GDNF compared to those in EGF alone (Figure 5A). Moreover, the growth over a one-month period confirmed that the addition of GDNF to a standard EGF-containing proliferation medium provides sustained augmentation of RPC numbers (Figure 5B).

### Caspase-3 activity

**Figure 6:**
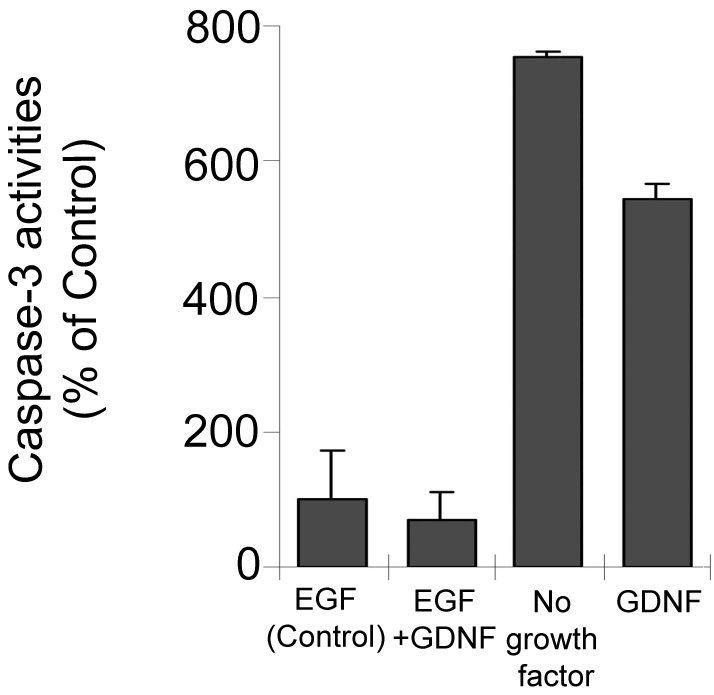
Change in caspase-3 activity. Cells were compared in terms of caspase activity under the same treatment conditions used previously, i.e., no growth factor, glial cell line-derived neurotrophic factor (GDNF), epidermal growth factor (EGF) and EGF+GDNF for 24 h. Experiments were performed in triplicate. Caspase-3 activity was evaluated spectrophotometrically at 405 nm in whole-cell lysates and calculated by construction of a para-nitroaniline calibration curve (data not shown). The y-axis represents percentages of caspase-3 activity related to EGF medium (control; n=10). Data are expressed as the mean±SD (p>0.05 versus EGF; p<0.05 versus EGF; p<0.05 versus no growth factor).

We determined the level of caspase-3 activity under conditions with or without EGF or GDNF after 24 h treatment. Caspase-3 activity levels with no growth factors, as well as with GDNF alone, were substantially elevated compared to conditions that included EGF (p<0.05), and activity was significantly higher in the no-growth-factor condition than in GDNF alone (p<0.05). Between EGF and EGF+GDNF, no difference was observed (Figure 6).

### Microarray screening for GDNF-induced changes in gene expression

**Figure 7:**
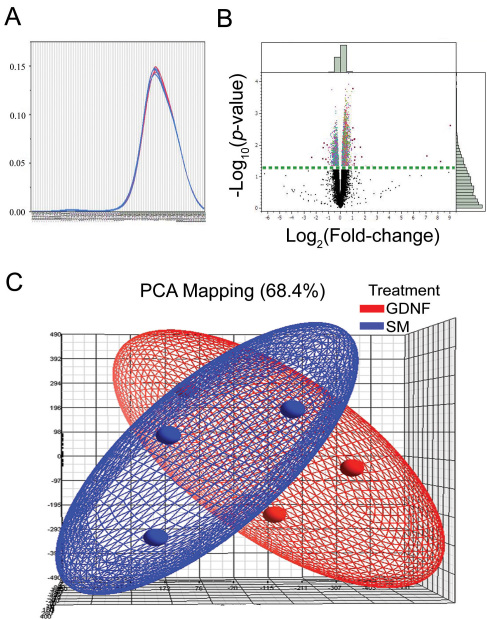
Overlayed kernel density estimates, principle component analysis, and volcano plot. **A**: Overlayed kernel density estimates show the raw univariate distributions for all arrays. The distributions for these arrays are very similar, indicating that this is a high-quality data set that should require little if any normalization for further analysis. **B**: Volcano plot from ANOVA analysis of the differential expression under epidermal growth factor (EGF) and EGF+glial cell line-derived neurotrophic factor (GDNF) conditions reveals differentially expressed genes. The fold change values between two conditions (log_10_ transformed) were plotted on the x-axis and were compared to the negative log_10_ transformed p values on the y-axis. Genes with a transformed p value of at least 0.05 and a transformed fold change of at least two in the upper left and upper right of the volcano plot are highlighted in red. Among these genes, eight are predicted genes and the other 16 are listed in Table 2. Histograms along the borders were generated via all detected genes. The dotted straight horizontal line represents the value 1.3 [-log_10_ (p value=0.05)], and the genes above that line are significantly different in expression level between the two conditions. The pale green bars in the histograms along the borders show numbers of all genes, and the dark green bars inside represent genes with a p value of <0.05. **C**: Principle component analysis (PCA) of gene expression signals reveals a broad degree of overlap in expression patterns between the two treatment conditions, with little suggestion of a treatment effect. Blue indicates data from retinal progenitor cells (RPCs) cultured in standard, epidermal growth factor-based medium (SM), while red indicates data from standard medium supplemented with GDNF. Individual microarrays (3 each condition) are indicated as small orbs and the general data distribution for each condition as an elliptical field of the corresponding color.

The signal levels of individual cDNA from the experimental (EGF+GDNF) and control (EGF) groups were compared after hybridization on a mouse whole-genome, gene-level microarray that consisted of over 28,853 genes. The fold change and p value of each of the genes were calculated, and log_2_ (fold change) and −log_10_ (p value) were transformed. Genes with a p value of <0.05 and an absolute fold change value of >1 were arbitrarily defined as upregulated in GDNF-treated cells, whereas those with a p value <0.05 and an absolute fold change value of <1 were defined as downregulated. The distribution analysis was done as a quality control step. The overlayed kernel density estimates (Figure 7A) derived from the distribution analysis showed the raw univariate distributions for all arrays, and allowed the visualization of sources of variation attributed to technical procedures. The distributions for these arrays were very similar, indicating that this represented a high-quality data set requiring little if any normalization for further analysis. Principle component analysis revealed a broad degree of overlap in expression patterns between the two treatment conditions, with little suggestion of a treatment effect (Figure 7C). However, ANOVA analysis showed that of the approximately 25,189 genes that were expressed in the cultures treated with EGF+GDNF, 5,250 of these were associated with the presence of GDNF (Figure 7B). A total of 3,865 genes were upregulated and 1,385 genes down regulated following the addition of GDNF to EGF-containing standard medium. In this case, changes in 24 genes were detected in the EGF+GDNF condition (Table 2). Among those were 16 known and eight predicted genes, the latter not yet associated with any biologic functions (data not shown). Of the 16 known genes, six were olfactory receptors with three being upregulated and three down-regulated. There were two genes with possible associations to photoreceptor cilia, namely myosin IXa and a dynein (Dnahc7a); however, changes were slight and in opposite directions.

**Table 2:** Genes detected by microarray screening as exhibiting significant changes in expression level following treatment of murine retinal progenitor cells (RPCs) with glial cell line-derived neurotrophic factor (GDNF).

Gene symbol*	**Gene accession**	**Gene description**	**GO_Biological_Process_Term**	**log2 (fold change)§**	**-log10 (p-value)#**
Olfr988	NM_001011534	olfactory receptor 988	signal transduction;G-protein coupled receptor protein signaling pathway; sensory perception of smell	2.6	1.32
Olfr444	NM_146656	olfactory receptor 444	signal transduction;G-protein coupled receptor protein signaling pathway; sensory perception of smell	−2.39	1.62
Tex12	NM_025687	testis expressed gene 12	—	2.04	1.33
Dnahc7a	XM_001473948	dynein, axonemal, heavy chain 7A	biological_process	1.73	1.63
Olfr1290	NM_146262	olfactory receptor 1290	signal transduction ;G-protein coupled receptor protein signaling pathway; sensory perception of smell	−1.45	1.58
Olfr860	NM_146528	olfactory receptor 860	signal transduction;G-protein coupled receptor protein signaling pathway; sensory perception of smell	−1.43	1.92
Ear1	NM_007894	eosinophil-associated, RNase A family, member 1	—	−1.22	1.56
Olfr486	NM_146496	olfactory receptor 486	signal transduction;G-protein coupled receptor protein signaling pathway; sensory perception of smell; response to stimulus	1.19	1.63
BC021785	ENSMUST00000095749	cDNA sequence BC021785	carbohydrate transport	1.18	2.19
Vmn2r23	NM_001104638	vomeronasal 2, receptor 23	—	1.16	1.94
H2-M10.5	NM_177637	histocompatibility 2, M region locus 10.5	—	1.16	1.52
Olfr1221	NM_146902	olfactory receptor 1221	signal transduction;G-protein coupled receptor protein signaling pathway; sensory perception of smell	1.15	1.34
Dsg1b	NM_181682	desmoglein 1 beta	cell adhesion;homophilic cell adhesion	1.13	2.17
Mafg	AK047224	v-maf musculoaponeurotic fibrosarcoma oncogene family, protein G (avian)	in utero embryonic development; transcription; regulation of transcription, DNA-dependent;adult behavior;regulation of cell proliferation;regulation of epidermal cell differentiation	−1.11	2.02
Fcrls	NM_030707	Fc receptor-like S, scavenger receptor	—	1.02	3.79
Myo9a	ENSMUST00000085572	myosin IXa	—	−1	1.47

A combination of *p*-value<0.05 [equals that –log_10_(*p*-value)>1.3] and absolute value of fold change≥2 [equals the absolute value of log_2_(fold change)≥1] were applied. Totally 24 genes were detected and eight of them are predicted genes for which no biologic functions are known so far (data not shown). *: Gene symbol (Gene name; Affymetrix probe set identification) **§**: log_2_(fold change), “#”: “minus values represent the genes that were down regulated in epidermal growth factor (EGF)+ Glial cell line-derived neurotrophic factor (GDNF) compare EGF. Condition of GDNF #: -log_10_(p-value), p-value, log_10_ transformed.

### Effect of GDNF on RPC gene expression determined by qPCR

**Figure 8:**
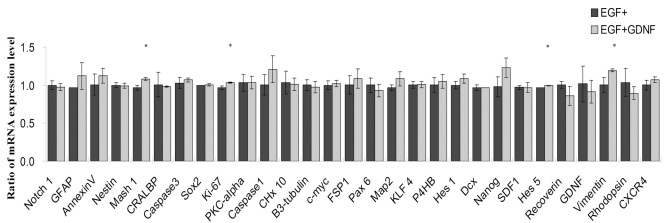
Effect of the glial cell line-derived neurotrophic factor (GDNF) on gene expression profile of retinal progenitor cell (RPCs) evaluated by qPCR. After 5 days of culturing in either the epidermal growth factor (EGF) alone or in EGF+GDNF conditions, the expression of the selected progenitor through retinal and apoptosis markers was evaluated by quantitative reverse-transcription PCR. For each gene, expression levels in the EGF-alone condition (black) were set to 1.0, and the relative expression in the EGF+GDNF condition (gray) was expressed proportionately. Expression levels of the progenitor markers *Nestin*, *Sox2*, *K_i_-67*, *Chx10*, *C-myc*, and *Vimentin* were sustained with the addition of GDNF, suggesting that the progenitor phenotype is not negated by exposure to this cytokine. Similarly, expression levels of the precursor and lineage-related markers *CRALBP*, *PKC-α*, *βIII-Tubulin*, *MAP2*, *DCX*, and *Recoverin* were not significantly affected by the addition of GDNF to EGF-based proliferation medium. However, there were small but statistically significant increases in the expression of *Vimentin* (1.20 fold), *Mash 1* (1.12 fold), *K_i_-67* (1.07 fold), and *Hes5* (1.03 fold) versus the same gene under EGF-alone conditions (*p<0.05). The x-axis shows different genes; the y-axis shows ratios of mRNA expression levels for the treatment groups.

In addition to checking for global changes, the potential influence of GDNF on a specific subset of genes with known associations to RPCs was also evaluated using quantitative PCR. Following treatment of RPCs with GDNF for up to 5 days, expression of most progenitor-associated markers remained effectively stable (Figure 8). At baseline, in the presence of EGF, cultured RPCs expressed the progenitor markers *Nestin*, *K_i_-67*, ceh-10 homeo domain containing homolog (*C. elegans*) [*Chx10*], sex determining region Y-box 2 *(Sox2)*, v-myc myelocytomatosis viral oncogene homolog (avian) [*C-myc*], and *Vimentin*. This pattern was sustained in the EGF+GDNF condition. Similarly, the expression levels of precursor and lineage-related markers including βIII-tubulin, microtubule-associated protein 2 (*MAP2*), doublecortin (*DCX*), cellular retinaldehyde binding protein (*CRALBP*), protein kinase C, alpha (*PKC-α)*, and *Recoverin*. These were relatively unaffected by the addition of GDNF to the EGF-based proliferation medium (Figure 1). Of the above markers, a subset exhibited marginally increased expression, namely, Hes5 (1.03-fold), *K_i_-67* (1.07-fold), *Mash 1* (1.12-fold) and *Vimentin* (1.20-fold; Figure 8). Of these, the microarrays showed no significant difference for *Hes5*, *K_i_-67*, or *Vimentin* (*Mash 1* was not included; Table 3). Taking the comparison further, the qPCR data did not confirm the marginal changes seen with the microarrays for *Nanog*, *Notch1*, or *GFAP*.

**Table 3:** Expression of selected genes of retinal progenitor cell (mRPC) in epidermal growth factor (EGF) and EGF+ Glial cell line-derived neurotrophic factor (GDNF) conditions.

**Genes**	**Description**	**Microarray**	**RT–PCR**
*K_i_-67*	Cell cycle protein, proliferation marker	N	Upregulation (*fold* change 1.07)
Nestin	Intermediate filament	N	N
Sox2	Transcription factor	N	N
Hes1	Transcription factor	N	N
Hes5	Progenitor marker	N	Upregulation (*fold* change 1.03)
Notch1	Progenitor marker	Down regulation# (*fold* change 0.98)	N
Pax6	Paired box	N	N
CXCR4	chemokine (C-X-C motif) receptor 4	N	N
DCX	Doublecortin,neuroblast marker	N	N
Vimentin	Intermediate filament	N	Upregulation (*fold* change 1.20)
GFAP	Intermediate filament	Upregulation (*fold* change 1.10)	N
PKC-α	Bipolar neurons marker	N	N
Recoverin	Phototransduction-related protein	N	N
Rhodopsin	Rod photoreceptors marker	N	N
β3-tubulin	Microtubule protein	N	N
Caspase 1	Apoptosis marker	N	N
Caspase 3	Apoptosis marker	N	N
Annexin A5	Apoptosis marker	N	N
KLF4	Kruppel-like factor 4, induced pluripotent stem cells marker	N	N
Nanog	Nanog homeobox	Upregulation** (*fold* change 1.05)	N*
P4HB	prolyl 4-hydroxylase, beta polypeptide, Fibroblast Marker	N	N
GDNF	glial cell line derived neurotrophic factor	N	N

* N: No statistically significant difference was found between EGF and EGF+GDNF conditions. ** Upregulation: The expression of genes was upregulated in EGF+GDNF comparing EGF. # Down regulation: The expression of genes was down regulated in EGF+GDNF comparing EGF.

### Western blot analysis

PCR detects changes in mRNA expression; however, the extent to which these changes are reflected at the level of proteins is also of interest. Western blotting was employed for this purpose on samples from the EGF alone and the EGF+GDNF treatment groups. Nestin was used as a marker of neural progenitor cells and K_i_-67 as a marker of cell proliferation.

**Figure 9:**
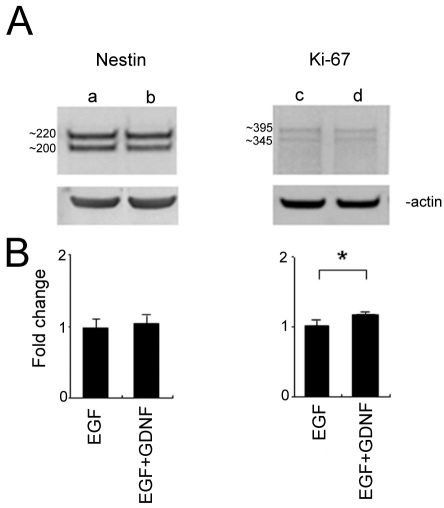
Western blot analysis of retinal progenitor cell (RPCs) cultured with either epidermal growth factor (EGF) or EGF+GDNF for 5 days. **A**: Anti-nestin identified two distinct protein bands with anticipated molecular weights of approximately 220 kDa and approximately 200 kDa. Two bands were also identified by anti-K_i_-67 with molecular weights approximately 395 kDa and approximately 345 kDa consistent with the molecular weights of alternatively spliced K_i_-67 isoforms [31]. Lane a: nestin in EGF; lane b: nestin in EGF+GDNF; lane c: K_i_-67 in EGF; lane d: K_i_-67 in EGF+GDNF. β-actin was used as the loading control (lower panels). B: Quantitative analysis showed no significant difference in nestin expression between treatment conditions. For K_i_-67, there was a statistically significant increase in total protein expression for the EGF+GDNF condition of 1.18±0.009 fold (*p<0.02).

Anti-nestin identified two bands with the anticipated molecular weight, approximately 220 kDa and 200 kDa, with β-actin labeling used as the loading control. The upper band of nestin (approximately 220 kDa) was strongly labeled in both the EGF and EGF+GDNF conditions and the lower band (approximately 200 kDa) was little changed in the EGF+GDNF condition, compared with EGF alone. Overall, no significant difference between treatment conditions was detected by quantification of the entire blot-lane intensity. Two bands were detected by K_i_-67 antibody, approximately 395 kDa and 345 kDa. Quantification analysis showed that the intensity of the entire blot lane was increased in the EGF+GDNF condition by 1.189±0.0095-fold (Figure 9). These results at the protein level were consistent with the mRNA results for *Nestin* and *K_i_-67*, as determined by qPCR (Figure 8), namely, that the total *Nestin* mRNA did not change in response to EGF+GDNF treatment over 5 days, whereas *K_i_-67* was slightly upregulated (Figure 9).

## Discussion

Cytokines and stem cells are both powerful biologic agents and feature in many regenerative strategies currently being investigated. While these two categories of agents have evident therapeutic potential in their own right, the possibility that complementary or synergistic effects could be achieved using a combinatorial approach is also worthy of attention. Of particular interest in the setting of retinal degeneration is the use of tissue-specific progenitors, namely RPCs, to deliver neurotrophic factors with known neuroprotective activity, a notable example being GDNF. It has been established that cultured central nervous system progenitor cells can be transduced to express a variety of transgenes [31-33], including GDNF [27,34]. Because the role of GDNF can differ between cell types and developmental stages, it is important to understand the effect of this factor not only on the retina, but also on potential donor cells such as RPCs. Here, we have shown that exogenous GDNF has several subtle yet discernable influences on cultured murine RPCs, including augmentation of EGF-induced proliferation as well as a modest reduction in apoptosis when EGF is withdrawn.

A growing body of work has shown that growth factors, alone or in combination, exert important effects on cellular behavior, particularly during development. For example, Zheng and colleagues [35] have shown that EGF, fibroblast growth factor, and hepatocyte growth factor all induce changes in fetal liver hepatocytes in vitro, and that different combinations of these growth factors show various effects on the proliferation and differentiation of the cells. Salient to the current report, Ahmad and colleagues have shown that retinal progenitors proliferate and remain undifferentiated in vitro in the presence of epidermal growth factor (EGF), and display properties similar to stem cells [36], as have we, having tested the same murine RPCs here [3]. Ahmad and colleagues [37] have also shown that EGF can mediate suppression of retinal rod photoreceptor differentiation. Although the influence of EGF and ciliary neurotrophic factor (CNTF) on RPCs has been reported, the influence of GDNF has not, to our knowledge, been previously investigated. The relative lack of response of murine RPCs to GDNF found in the present study is in contrast to what we have previously shown for other treatment conditions, namely serum as well as a different cytokine, CNTF [38]. Both serum and CNTF had notable differentiating influences on murine RPCs, whereas the present study indicates that GDNF does not, under comparable conditions. In the presence of EGF, exposure to GDNF appears to be compatible with maintaining the undifferentiated state of these cells.

GDNF has emerged as a particularly important cytokine by virtue of its powerful neuroprotective influence, as demonstrated in multiple models of neurodegeneration [39,40]. However, despite significant progress in the understanding of the GDNF signaling pathways and receptor interactions, the exact cellular mechanisms responsible for these neuroprotective effects are not yet fully understood. Furthermore, despite therapeutic promise in the clinical treatment of retinal and other diseases, this growth factor has yet to be validated in the treatment of patients. A major challenge to the use of cytokines is their rapid breakdown in vivo by endogenous proteases. Cell-based delivery of these molecules is attractive for this reason, and the present findings have implications for the development of GDNF-overexpressing RPCs.

In terms of the effects reported in the present study, a proliferative influence has been described previously, and it has been reported that GDNF is able to improve the survival and differentiation, both in vivo and in vitro, of a variety of brain neurons [41,42], as well as being essential for the proliferation of enteric precursor cells [43]. Similarly, GDNF has been found to stimulate proliferation during renal development [44]. Beyond this, a regulatory mitogenic effect has been demonstrated for rat glioma cells by adding exogenous GDNF or using antisense oligonucleotides for the suppression of endogenous GDNF. Moreover, a similar proliferative effect of GDNF has been established in photoreceptor-enriched rat monolayer cultures [45]. Our current data extend these findings to the in vitro behavior of RPCs, a cell type of great developmental significance in the retina. It remains possible that different, and potentially deleterious, effects could be produced by exposure of RPCs to GDNF at substantially higher concentrations, as might occur with very high overexpression of an otherwise therapeutic transgene.

Of importance to the development of GDNF-overexpressing RPCs, neither augmentation of proliferation nor reduction in apoptosis pose an impediment to the expansion of these cells in culture. This allays one potential concern that arises when contemplating the genetic modification of a proliferative cell type for cytokine delivery. In other words, it is important that a constitutively expressed therapeutic transgene not impede the proliferative ability of the carrier cell, thereby necessitating repeated re-derivation and genetic modification. For instance, CNTF has demonstrated potential as a neurotrophic agent, however, the known differentiating influence of this factor on RPCs could pose a hindrance to the use of this candidate transgene. It is also important that the potential therapeutic molecule not induce undesirable changes in the phenotypic status of the cell used for delivery. In this context, it is reassuring that the global gene expression of murine RPCs was not substantially altered by exposure to GDNF.

### Conclusions

This study shows that GDNF is not a potent inducer of RPC differentiation. Its results support the use of these cells for GDNF delivery to the diseased retina. Because the expression profile of cultured RPCs is not markedly altered by exposure to GDNF, it may be the case that transduced RPCs will retain their ability to repopulate local retinal populations following transplantation.

## Supplementary Material

Supporting Movie
